# Neuropsychiatric Disorders and Readmission Rates: A 10-Year Single-Center Retrospective Cohort Study at an Acute Psychiatric Facility in Abu Dhabi, UAE

**DOI:** 10.7759/cureus.111817

**Published:** 2026-06-30

**Authors:** Safana AlFardan, Sona Varghese, Hayat Abdulaziz, Ruqqia Mir, Alaa Haweel, Tarek Shahrour

**Affiliations:** 1 Azrieli Brain Medicine, University of Toronto, Toronto, CAN; 2 Psychiatry, Abu Dhabi Health Services, Abu Dhabi, ARE; 3 Psychology, Heriot-Watt University, Dubai, ARE; 4 Neurology, Abu Dhabi Stem Cells Center (ADSCC), Abu Dhabi, ARE; 5 Psychiatry, American Center for Psychiatry and Neurology, Abu Dhabi, ARE

**Keywords:** mental health hospitalization, multidisciplinary care, neuropsychiatric disorders, psychiatric readmission, united arab emirates (uae)

## Abstract

Background

Neuropsychiatric disorders, with their complexities, manifest with a blend of neurological and psychiatric symptoms that pose significant challenges within classification systems and their overall management. These intricate, perplexing disorders appear to contribute to increased readmission rates, illustrating the need for concrete evidence to facilitate the urgent and longstanding requisite for personalized care and research development within an interdisciplinary care framework.

Methodology

This retrospective cohort study analyzed a decade's worth of data from a 250-bed acute inpatient care facility in Abu Dhabi, United Arab Emirates. The dataset included a total of 334 patients: 75 with neuropsychiatric disorders and 259 with solely primary psychiatric disorders.

Results

As predicted, high readmission rates were observed in the neuropsychiatric disorder cohort in comparison to the primary psychiatric disorder cohort. Annual readmission rates averaged 0.37 for neuropsychiatric versus 0.16 for psychiatric cases alone. Furthermore, emerging trends of demographic disparities were identified, where the neuropsychiatric cohort demonstrated a high readmission frequency across various age, gender, and ethnic groups.

Conclusion

The readmission rates of neuropsychiatric disorders were substantially higher in the acute psychiatric care facility in Abu Dhabi, compared to primary psychiatric disorders. The results of this study in the acute, yet tertiary, inpatient psychiatric facility in Abu Dhabi highlight an urgent necessity for both specialized multidisciplinary care strategies in this vulnerable population and a specific focus on this population’s perpetuating unmet needs to improve their overall prognosis and decrease hospital readmission rates.

## Introduction

Neuropsychiatry is a complex interwoven discipline that exists at the interface of neurological and psychiatric disorders [1]. It often exhibits a blend of both brain and mind disorder features, due to the shared neuronal networks encompassed within the organ as a whole [1]. Post the 1960s, the deinstitutionalization shift and movement demonstrated notorious and profound impacts on individuals with “severe mental disorders,” where attempts at reintegration into society without established supportive frameworks led to difficulties in receiving holistic care and poorer prognosis [2]. Efforts to navigate within the realm of complex neuropsychiatric conditions can present challenges during their clinical crossings within fragmented systems, further complicating care and consequently leading to recidivism through the previously documented “revolving door” phenomenon [3].

Psychiatric disorder readmission rates are an important indicator of mental health system performance and overall effectiveness. It remains a frequent observation within international literature that annual hospital readmission rates due to psychiatric disorders range between 21% and 42% [4]. While documented in pediatric populations [5], the contribution of neurological comorbidities to these readmissions has not been satisfactorily documented in adult populations. One systematic review did, however, illustrate higher readmission rates in patients with dementia and comorbid depression, demonstrating the essential requirement for deeper investigation within neuropsychiatric research [6].

Recent studies emphasize that patients with a blend of cognitive, behavioral, and emotional dysregulation are more likely to fall through the cracks of the current fragmented networks, leading to discontinuity of care [1,7]. Within this regional context, sociocultural factors are also relevant, although directly comparable adult neuropsychiatric data remain limited. There is also emerging data within the Middle East, particularly with the Gulf Cooperation Council (GCC) countries, that patients with neuropsychiatric disorders are disproportionately subject to historically primed sociocultural factors with public and healthcare personnel-related stigma [8] (note: Reference [8] is a protocol focused on adolescents and is not direct adult neuropsychiatric evidence; it should be replaced with completed regional adult data where available). This plays into the conundrum of increased demand for mental health services with limited supply, given the limited psychosocial framework support offered, consequent to the sheer fragmentation and scarcity of accessible allied health and community resources. Nonetheless, the field continues to appreciate the bidirectional relationship between neurological and psychiatric disorders, and the requirement for an integrated and comprehensive focused care approach is becoming increasingly recognized, in efforts to improve health outcomes along with patient and caregiver-related quality of life.

The proposed study addresses this gap in the literature by examining the rates of readmission for patients with neuropsychiatric disorders in comparison to those with only primary psychiatric disorders at an acute inpatient care facility in Abu Dhabi, UAE. The study examines data from a 250-bed acute care facility and provides an in-depth analysis that spans over 10 years (2009-2019). It is comprised of 334 patients: 75 with defined neuropsychiatric disorders and 259 with primary psychiatric disorders. This study identifies the key demographic and clinical characteristics associated with readmission in this inter-related group, with the aim of informing more individualized and sustainable management plans and reducing the burden on healthcare and psychosocial systems.

## Materials and methods

Study design

A retrospective cohort study was carried out to determine the impact of neuropsychiatric disorders on readmission rates at Sheikh Khalifa Medical City (SKMC), an acute psychiatric facility in Abu Dhabi, UAE. Data collected were over a set 10-year timeframe: January 2009-January 2019. This allowed for evaluation of the long-term readmission trends and permitted further exploration into the stratified impact of neuropsychiatric disorders vs. primary psychiatric disorders on the healthcare system.

Study population

The target population included adult patients (≥18 years) admitted to the acute psychiatric institution within Abu Dhabi between January 2009 and January 2019, who experienced at least one readmission within 30 days and one year of index admission. Diagnostic profiles of the patients were selected and confirmed through extensive sifting within lengthy medical records, and patients were placed into two categories. The neuropsychiatry category (Neuropsych), in which patients were diagnosed with both a neurological and a psychiatric disorder, and the primary psychiatric category (Psych), in which the patients did not have an accompanying neurological disorder. The eligibility requirement was satisfied by 334 patients for this study, with 75 in the Neuropsych group and 259 in the Psych group. Because eligibility required at least one documented readmission, the analytic sample was conditioned on the readmission outcome; the proportions reported in this study therefore describe the distribution of readmissions within an already-readmitted cohort rather than the incidence or risk of readmission across all index admissions, and the between-group figures should be interpreted as relative readmission burden rather than true population readmission rates.

Inclusion criteria

Inclusion criteria for the study required participants to be 18 years or older at the time of their first admission and to have at least one documented readmission within the study outcome period. For inclusion within the Neuropsych group, patients had to have confirmed diagnoses of both psychiatric and neurological disorders, while those in the Psych group had to have psychiatric diagnoses without documented or clear neurological comorbidities.

Exclusion criteria

Exclusion criteria included patients with only a single admission (i.e., no readmissions) during the study period, those under the age of 18 at the time of initial admission, and patients with incomplete medical records, particularly those missing diagnostic details, readmission dates, or treatment outcomes.

Data collection

Data were extracted from the electronic medical health record (EMHR) system, which includes detailed patient information such as demographics, ICD-9 and ICD-10 diagnosis codes, admission/discharge dates, and readmission data. Data extraction focused on identifying eligible patients and capturing relevant variables related to diagnosis and readmission events.

Study variables

A neuropsychiatric disorder, as determined by the ICD-9 and ICD-10 diagnostic codes recorded in the medical records of the patients [9,10], was the major exposure variable in this study. For this study, a neuropsychiatric disorder was operationally defined as a documented primary psychiatric diagnosis co-occurring with a confirmed neurological diagnosis (e.g., dementia, traumatic brain injury, Parkinson's disease, multiple sclerosis, or migraine-related disorders). Given the clinical heterogeneity of this composite category, diagnostic subgroups are reported by frequency in Table 1, and estimates for the pooled neuropsychiatric group should be interpreted with this heterogeneity in mind. The main outcome of interest was the one-year readmission status, which was a binary variable that defined whether a patient was readmitted within one year after discharge. An additional minor outcome, the 30-day readmission rate, was also evaluated to obtain short-term readmission trends.

**Table 1:** Most frequent diagnoses and associated readmission proportions, by cohort.

Psych diagnosis	Readmission rate (%) for Psych	Neuropsych diagnosis	Readmission rate (%) for Neuro
Alcohol-induced psychotic disorder with hallucinations	100.00%	Multiple sclerosis + migraine	100.00%
Adjustment disorder	56.25%	Non-psychotic post-traumatic stress disorder	100.00%
Bipolar disorder	34.48%	Parkinson's disease	100.00%
Borderline personality disorder	33.33%	Cognitive decline; vascular + Alzheimer's dementia	50.00%
Depressive psychosis	25.00%	Alzheimer's dementia	44.44%

To adjust to the possibility of confounding factors that could affect the risk of readmission, several variables were taken into consideration. These were demographic (age, sex), clinical (illness severity, assessed via the Clinical Global Impression-Severity (CGI-S) scale) [11,12], and more global contextual (socioeconomic status (SES), living situation, and insurance coverage) factors. Moreover, the Charlson Comorbidity Index (CCI) was used to ensure that the effect of other medical complications on the outcome of readmission was appropriately controlled for during the analysis [13]. The CGI-S scale applied here is described in the Guy (1976) ECDEU Assessment Manual for Psychopharmacology [11].

Sample size determination

**Figure 1:**
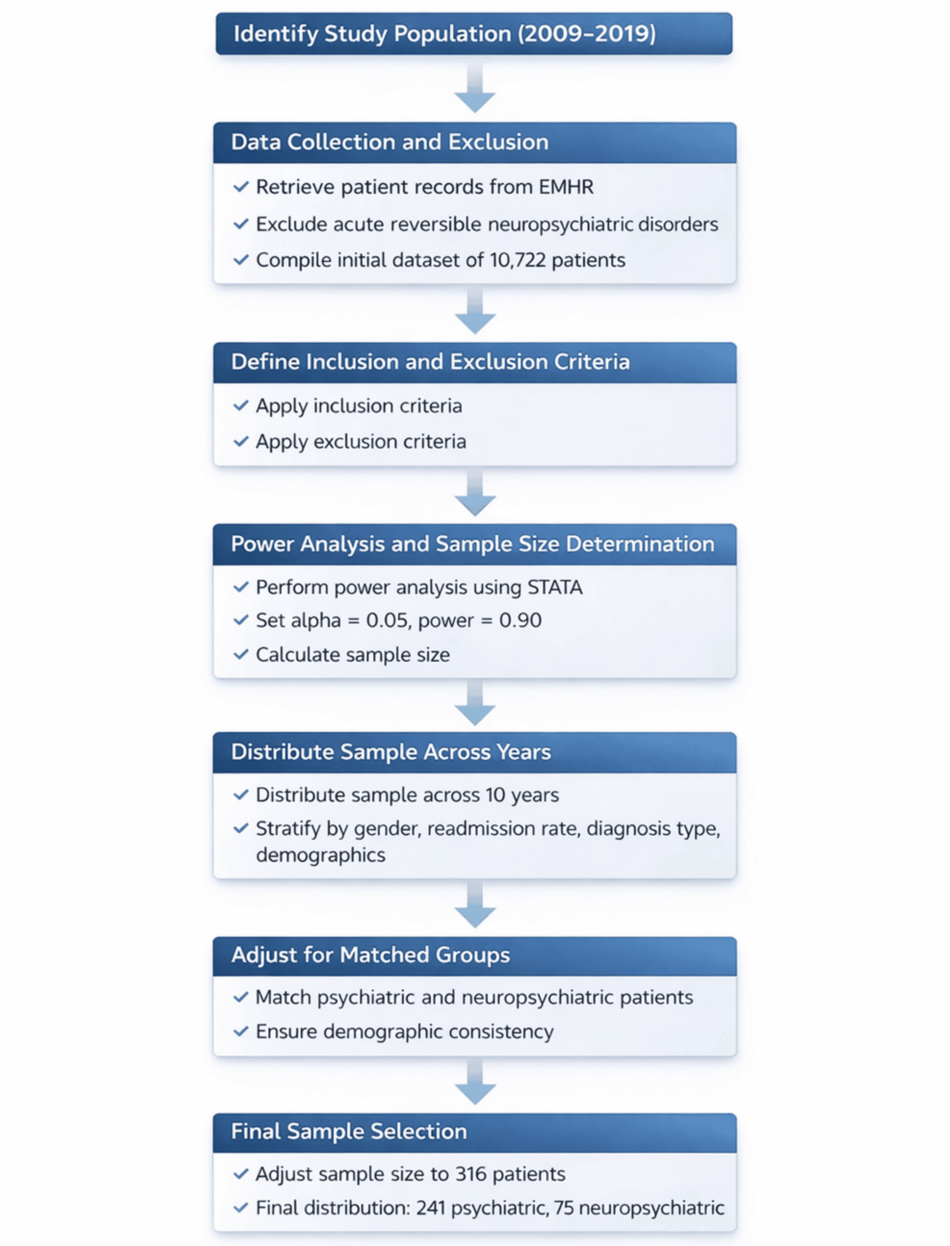
Patient selection and sample-size derivation flow diagram. EMHR: electronic medical health record

Sample size was calculated using STATA software (StataCorp LLC, College Station, TX, USA) based on a power analysis for a test of two proportions, applying a desired power of 90%, a significance level (α) of 0.05, and an assumed group ratio of Neuropsych to Psych of 1:9. Based on these parameters, the optimal total sample size was estimated to be 334 patients-75 in the Neuropsych group and 259 in the Psych group-providing sufficient power to detect statistically significant differences between the two groups, as shown in Figure 1.

Statistical analysis

Analysis was performed utilizing STATA and Python software (Python Software Foundation, Fredericksburg, VA, USA). Demographic data and frequencies of readmission were summarized using descriptive statistics with bar and pie charts. A time series analysis was implemented to study the trends of readmissions during the study period. Significant differences in the readmission rates between groups were analyzed using p-values. Because the primary outcome (one-year readmission) was binary, a binary logistic regression model was fitted to adjust for confounding factors and to quantify the association between diagnosis type and readmission. Associations are reported as regression coefficients with corresponding p-values; no hazard ratios were estimated, as no time-to-event (survival) analysis was performed.

Ethical considerations

The study adhered to all ethical guidelines for retrospective research. Institutional Review Board (IRB) approval was obtained prior to data collection. All patient data were anonymized to maintain confidentiality, and data handling was conducted in compliance with the health facility’s data protection policies and applicable regulations.

## Results

The results of this study highlight key differences in readmission rates between patients with neuropsychiatric disorders and those with primary psychiatric conditions within the acute psychiatric care setting.

Readmission rates

**Figure 2:**
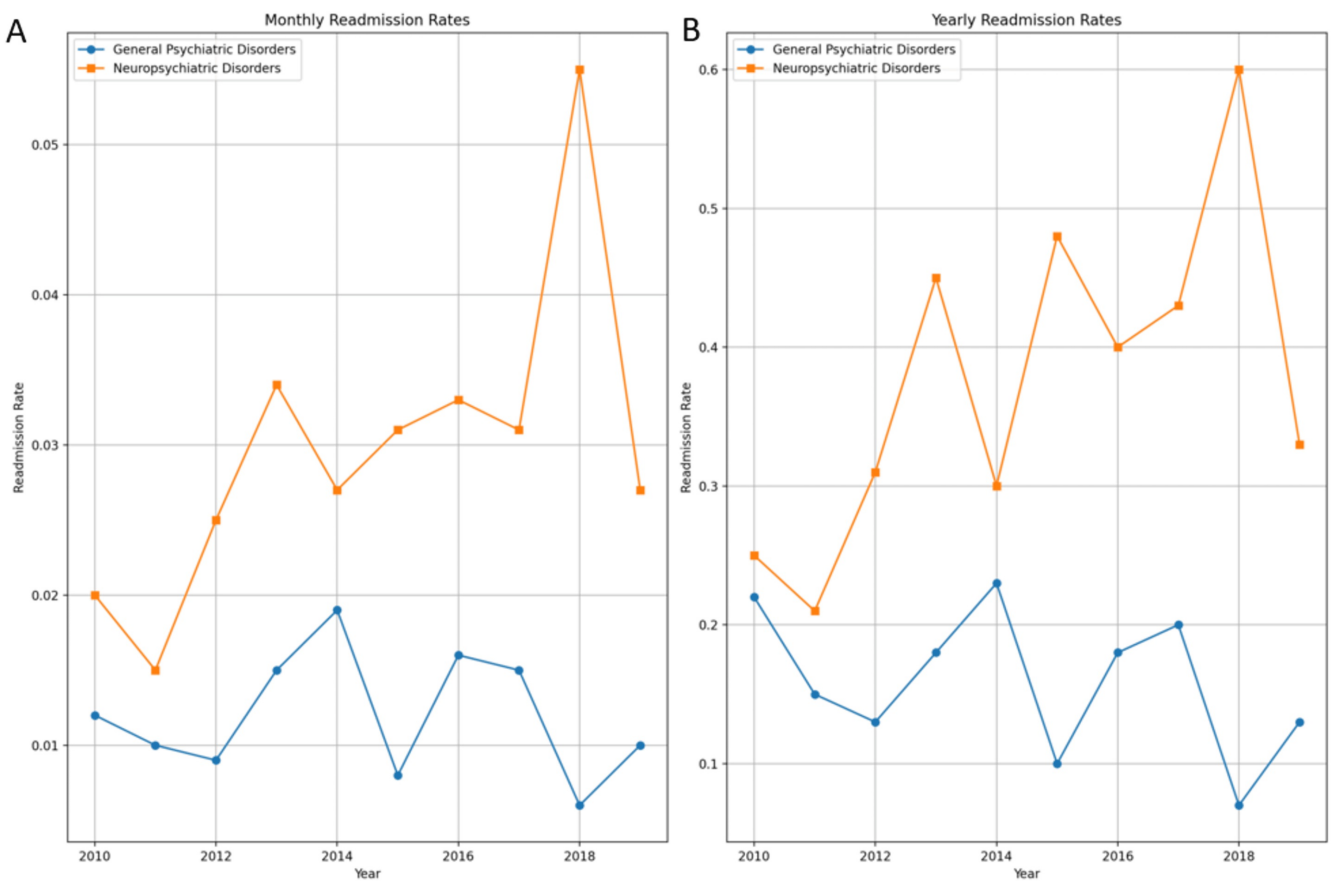
(A, B) Annual and monthly readmission proportions for the Neuropsych and Psych cohorts (2009-2019).

Figure 2 illustrates the annual and monthly readmission rates for neuropsychiatric and primary psychiatric disorders over the 10 years. According to the findings, neuropsychiatric disorders consistently exhibited a higher readmission rate compared to their primary psychiatric counterparts. Specifically, the annual readmission rates for neuropsychiatric patients were quite variable, reaching a peak of 5/9 (55.6%) in 2018. In contrast, the readmission rates for primary psychiatric disorders remained largely stable, averaging between 10.5% and 21.7% throughout the study period.

Demographic characteristics

Within the (Psych) group, given the multinational context of the UAE and the potential for confounding factors otherwise unaccounted for, nationality was stratified in a binary fashion and illustrated that the sample subset included 150 (62.2%) of Emirati patients, which was found to be significantly higher than non-Emirati patients (91 (37.8%)). Age was stratified into three categories and revealed that within the Psych group, 161 (66.8%) of those readmitted belonged to the 18-37 age group. Those in the 38-50 and above-50 age range groups included fewer patients and represented 42 (17.4%) and 38 (15.8%) of the total sample, respectively. Gender distribution displayed that the number of male patients (142 (58.9%)) was higher than the number of female patients (99 (41.1%)).

**Figure 3:**
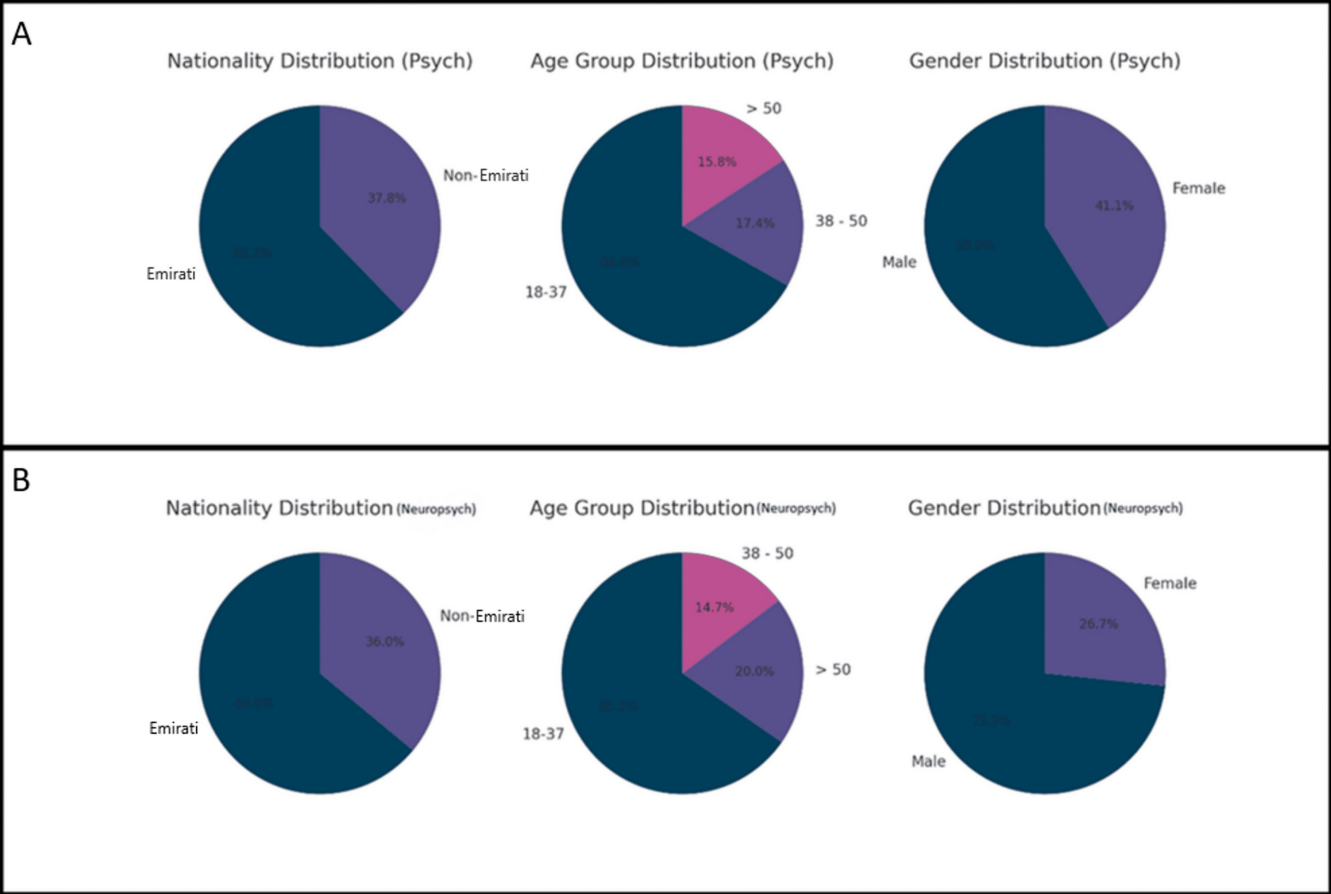
(A, B) Basic demographic characteristics of the Psych and Neuropsych cohorts (within-group percentages; descriptive only).

However, the demographic distribution of the Neuropsych group comprised 48 (64.0%) Emirati nationals vs. 27 (36.0%) represented as non-Emirati UAE patients. Aligning with expectations regarding age, the majority once again fell within the 18-37 year range (49 (65.3%)), followed by those aged above 50 at 15 (20.0%) and those between 38 and 50 years at 11 (14.7%). Gender-wise, males dominate the group at 55 (73.3%), with females representing 20 (26.7%). This indicates a predominantly young, male, and Emirati demographic within the Neuropsych category (Figure 3).

Multinomial logistic regression analysis: focused variables

Although none of the variables were found to be statistically significant, binary logistic regression of the impact of the selected confounding factors on the readmission rates between the neuropsychiatric and primary psychiatric disorder groups revealed a few exploratory trends. Considering that this was an exploratory study with a relatively small sample size, the following variables were grouped due to theoretical significance, effect size, and associated clinical implications, as indicated in Table 2. As none of these associations reached statistical significance, the observations below are exploratory only and should not be interpreted as evidence of true or causal associations; the directions of effect may reflect small sample size, measurement error, or selection artifact, and the language used here is intentionally tentative. Patients who lived alone or those with weak psychosocial support systems presented a positive coefficient of 0.79 (p = 0.459), which may be consistent with a greater risk associated with readmission. Although not statistically significant, this variable had one of the highest effect sizes in the analysis. Ever a well-proven risk factor in psychiatric literature, social isolation also proves to be the focus of any well-placed intervention strategy.

**Table 2:** Binary logistic regression of confounding factors and 1-year readmission (exploratory; no predictor reached statistical significance).

Variable	Coefficient	p-value	Reason for retention
Living alone/limited psychosocial support	0.79	0.459	Moderate positive effect size, aligns with known social risk factors for readmission.
Compliance with psychiatric medications	-0.54	0.538	Moderate protective trend; clinically important and modifiable.
Substance misuse/dependence	-0.9	0.289	Unexpected direction, but large effect size. Worth further exploration.
Socioeconomic status	-0.18	0.253	Social determinant; relevant in psychiatric readmission models.
Number of medications	-0.25	0.131	Shows a notable trend; easy to measure and may reflect treatment complexity.
Neurological Impairment Scale (NIS)	0.11	0.236	Neurological burden likely impacts readmission; small effect but relevant in the neuropsychiatric population.

Non-compliance with psychotropic agents was negatively related to readmission risk, which displayed a coefficient value of -0.54 (p = 0.538). Interestingly, and contrary to clinical expectations, this value could represent measurement or reporting biases. Nevertheless, medication adherence is a more tangible modifiable factor within psychiatric care settings and ought to be the focus of future investigation. Surprisingly, the coefficient of substance misuse/dependence was -0.90 (p = 0.289), which elucidated a possible decrease in the risk of readmission with these patients. This paradoxical pattern could be shaped by external systems of care (e.g., substance treatment programs), selection biases, or diagnostic misclassification. Although the direction of the effect is not desirable, the size of the coefficient is relatively large, which implies that this variable should be examined further.

Lower SES disclosed a negative coefficient of -0.18 (p = 0.253), indicating a lower risk for readmission. Although it does not correspond with a large proportion of the available literature, where lower SES is commonly associated with increased healthcare utilization, it could indicate underutilization or systematic hindrances to care re-evaluation. The SES role continues to be critical in illuminating health outcomes. The more medications, the lower risk of readmission; with a coefficient value of -0.25 (p = 0.131), this variable could be related to the criteria associated with admission to psychiatric facilities and the management of patients with a more complex medical profile in more medically adept inpatient settings. Although it was not found to be statistically significant, the effect size and its easily modifiable potential make this a handy variable to be mindful of within future analyses where holistic integrated care systems may further scrutinize and advise on medication regimens within the index psychiatric-related admissions. Furthermore, the coefficient of the Neurological Impairment Scale (NIS) score was positive, but small (0.11, p = 0.236), presenting a minor increase in the probability of readmission as neurological impairment increased [14]. This trend is clinically reasonable and consistent with the current literature on functional limitations and healthcare use generally.

Some factors displayed theoretical significance and therapeutically meaningful patterns, regardless of their predicted statistical significance. Further scrutiny of factors such as substance misuse, living alone, neurological dysfunction, polypharmacy, SES, and adherence to mental treatment is warranted. The results express that in order to validate and expand on this initial and more concrete foundation, a more thorough study with improved measurement precision is required. To better understand these complex relationships, prospective projects may also consider including qualitative data or patient/caregiver narratives with the use of previously validated scales for global comparison.

Readmission rates by age, gender, and severity

The readmission rates for neuropsychiatric and psychiatric patients of different ages, genders, and CGI-S levels are contrasted in Table 3. Notably, compared to psychiatric patients, neuropsychiatric patients had a higher readmission rate across the board. For instance, the readmission rate was 20% for psychiatric patients and 66.67% for neuropsychiatric patients in males aged 18-37 with a CGI-S of 5. Similarly, the readmission rate was considerably greater for neuropsychiatric patients between the ages of 38 and 50 and females over 50 with high CGI-S ratings.

**Table 3:** Readmission proportions stratified by age group, sex, and CGI-S in the Psych versus Neuropsych cohorts (exploratory). *"Not estimable": OR cannot be calculated because the Psych cell count is 0; report as "NE" in the table. Fisher's exact test used due to small cell sizes. OR: odds ratio; CI: confidence interval; NE: not estimable (zero cell); CGI-S: Clinical Global Impression-Severity

Age group (years)	Gender	CGI	% readmission Psych	% readmission Neuropsych	OR (95% CI)	p-value
18-37	Female	4	0	0.3333	Not estimable*	0.083
18-37	Female	5	0	0.2857	Not estimable*	0.2
18-37	Male	3	0.286	0.3333	1.25 (0.30-5.77)	1
18-37	Male	4	0.364	0.3333	1.62 (0.50-5.47)	0.51
18-37	Male	5	0.2	0.6667	8.00 (0.49-60.09)	0.242
38-50	Female	4	0	1	Not estimable*	0.091
38-50	Male	5	0	0.5	Not estimable*	0.222
>50	Female	5	0	0.6667	Not estimable*	0.143
>50	Male	5	0	0.4	Not estimable*	1

Comparison of factors between groups

The comparison of a number of parameters in the Neuropsych and Psych groups is presented in Table 4. The NIS scores of the neuropsychiatric patients were more widely distributed than those of the psychiatric group, with some of the patients scoring above the range (e.g., NIS = 17 or 23). The distribution of the CCI also showed that, unlike the neuropsychiatric group, which showed a more even distribution, the psychiatry patients showed a tendency to a lower index of comorbidity, of which most obtained a score of 1. The SES of the two groups, their living conditions, including high expressed emotions within family settings, polypharmacy, and use and abuse/dependence of psychotropic agents, were also distinct, implying dissimilar clinical and social parameters.

**Table 4:** Between-group comparison of clinical and psychosocial factors (Psych versus Neuropsych). NIS is structural (Psych patients have no NIS by definition). NIS was not applicable to the Psych group, as neurological impairment is a defining criterion of the Neuropsych group; no between-group statistical comparison was performed. Chi-squared test used for categorical variables; Mann-Whitney U test used for ordinal variables. Significance threshold: p < 0.05. NIS: Neurological Impairment Scale; CCI: Charlson Comorbidity Index

Variable	Test	Test statistic	p-value
NIS	Descriptive only	-	-
CCI	Mann-Whitney U	U = 10,171.5	0.001
Socioeconomic status	Mann-Whitney U	U = 8,191.5	0.027
Living alone/psychosocial support	Chi square	χ²(1) = 0.000	1
High expressed emotion at home	Chi square	χ²(1) = 47.962	<0.001
Number of medications	Mann-Whitney U	U = 14,106.0	<0.001
Compliance with psychiatric medications	Chi square	χ²(1) = 14.177	<0.001
Compliance with neurological medications	Chi square	χ²(1) = 129.931	<0.001
Compliance with the rest of the medications	Chi square	χ²(1) = 5.127	0.024
Substance misuse/dependence	Chi square	χ²(1) = 2.341	0.126

Diagnosis and readmission rates

The most common diagnoses and readmission rates for patients with mental and neuropsychiatric disorders are detailed in Table 1. The greatest readmission rate was 100% among psychiatric patients with alcohol-induced psychotic disorder, followed by adjustment disorder (56.25%) and bipolar disorder (34.48%). On the other hand, readmission rates were 100% for neuropsychiatric patients with Parkinson's disease, traumatic brain injury, multiple sclerosis, and migraine disorders. More complex neuropsychiatric disorders reflected high readmission rates linked to patients with cognitive decline, with vascular and Alzheimer's dementia in the lead.

The economic impact of psychiatric and neuropsychiatric recidivism is substantial and continues to place a significant burden on healthcare systems globally. In the United States, the average 30-day readmission cost for mood disorders is approximately $7,200, and for schizophrenia, it rises to $8,600, often surpassing the cost of the initial hospitalization [15]. Beyond direct medical costs, recidivism among patients with psychiatric conditions can also contribute to increased criminal justice system expenditures. These figures underscore the importance of identifying and addressing modifiable risk factors for readmission, not only to enhance clinical outcomes but also to alleviate the financial burden on public health systems.

## Discussion

Toward holistic neuropsychiatric care: implications for Brain Medicine

This study demonstrates a significant and overall sustained trend. Readmission rates of those with neuropsychiatric disorders are considerably higher in comparison to those with primary psychiatric disorders alone. This adds evidence to the scarce yet rising literature that urges more of a multidisciplinary, holistic, and integrated approach to the treatment of these disorders that are “brain-based.” The treatment of neuropsychiatric disorders with an integrated approach through an interdisciplinary lens outmaneuvers the classical models of practice with improved accessibility to superior care, thus leading to better overall health outcomes that would act to decrease recidivism within such settings. The concept of the dualistic approach to brain care must be both redefined and refined utilizing the interdependent biology, circuitry, and functionality of the brain; where neurological, cognitive, emotional, and behavioral symptoms are treated simultaneously amid a multipronged approach. This conceptual model has been adopted, piloted, and operationalized by the University of Toronto by Levitt et al. [16]. Similarly, Harvard Medical School has more recently embraced the "Brain Medicine" integrated care approach, which emphasizes the level of brain discipline expert “cross-talk” required for the provision of most informed holistic care, which is both “cost and quality saving” in the long run.

The demonstration by this study of readmissions of patients with neuropsychiatric disorders of all degrees of impairment, sex, and age highlights the need for diagnostically responsive and adaptable clinical systems within the health ecosystem [17,18]. Complex neuropsychiatric disorders, as exhibited, are underserved within fragmented systems, contributing to increased overall cost to global budgets afforded to hospitals and health [16]. A co-managed, integrated model of Brain Medicine would encourage all-rounded care by brain-related specialties [19]. It would also involve rehabilitation approaches of functional recovery, including cognitive retraining, psychopharmacology, and psychosocial therapy [20].

Clinical significance and the post-COVID-19 context

The findings of the studies are also to be contextualized within the pre-COVID era (2009-2019). After the outbreak of the COVID-19 pandemic, studies have suggested potential risks for decompensation in the management of chronic neurological illnesses [21]. There is some evidence that patients with pre-existing neuropsychiatric conditions, especially those with neurodegenerative diseases or traumatic brain injuries, were at increased risk of higher relapse rates, social isolation, and diminished access to services during and after the pandemic, all of which are likely to increase the risk of readmission [22].

Similarly, factors contributing to the overall allostatic load, such as unemployment, recent bereavement, and health anxiety, were magnified during the pandemic. It is anticipated that this could have a disproportionately negative impact on those with complex neuropsychiatric conditions. Recent studies suggest remote assessment tools can be feasible for cognitive screening, though challenges remain for comprehensive in-person neuropsychiatric assessments [23]. This post-COVID-19 phenomenon may indicate that readmission rates in neuropsychiatric populations could be greater than observed in this pre-pandemic dataset; this is offered as a hypothesis, as the previously cited references do not directly support post-pandemic readmission trends in neuropsychiatric populations and should be replaced with continuity-of-care or readmission studies in this population.

Therefore, health systems, particularly those in rapidly developing areas such as the UAE, ought to expect growing neuropsychiatric care demands and increased complexity in holistic healthcare delivery. The challenges could be addressed in future crises by strengthening continuity-of-care procedures, improving accessibility to care, specifically with neurorehabilitation services, and integrating flexible, interdisciplinary response teams.

Alignment with clinical data and future directions

The results provide evidence for the hypothesis that neuropsychiatric disorders are associated with increased risk of decompensation and readmission, even after controlling for medication adherence and comorbidities. Though regression models were not statistically significant predictors, possibly owing to sample size limitations, emerging trends suggest that social determinants (e.g., environmental living conditions and emotional climate) and treatment adherence continue to be important indicators. These findings provide evidence for patient-centered models of care that integrate mental health services, neurological treatment, and social support within a coordinated model.

Given the heterogeneous population base and multiplanar manifestation of disorders such as Parkinson's disease, cognitive impairment, epilepsy, and traumatic brain injury, future studies would have to examine stratified models of care tailored to specific neuropsychiatric subtypes. Prospective multicenter studies throughout the Gulf region are also an absolute necessity to validate these results and establish care protocols that are both reliable and scalable.

Study strengths and limitations

This study’s strengths lie in its retrospective nature, and a comprehensive analysis was performed with data collected over a 10-year timeframe. This serves as a fair starting point to explore trends and outcomes in complex neuropsychiatric care. However, it is not without its respective caveats. Some limitations include that it is prone to patient and healthcare personnel recall bias, and a lack of inter-rater reliability within measuring aspects further plays into this, given the lengthy examination protocols adopted while examining available EMHR. Moreover, using ICD coding to specify diagnostic categories may also lead to variability and diagnostic misclassification when defining patient cohorts, a feature notoriously documented within neuropsychiatric literature. The absence of a control center could also be regarded as a weakness in extrapolating results to the UAE as a whole or other healthcare environments.

Most importantly, eligibility required at least one prior readmission, so the cohort was conditioned on the outcome; this selection mechanism-rather than recall or coding bias-is the principal threat to validity, because it precludes estimation of true readmission incidence or risk in the source population and may distort the between-group comparison. This limitation should be considered when interpreting all readmission figures reported above.

## Conclusions

This study demonstrates substantially higher readmission rates for patients with neuropsychiatric disorders compared to those with primary psychiatric disorders in the acute inpatient psychiatric setting of Abu Dhabi. Mean annual readmission rates for neuropsychiatric disorders exceeded those of psychiatric disorders by more than twofold (0.37 vs. 0.16), with consistent trends observed in monthly rates (0.03 vs. 0.01).

These findings underscore the complexity of neuropsychiatric case management and highlight the urgent need for individualized, multidisciplinary care strategies, robust post-discharge follow-up, and targeted policy reforms to reduce the disproportionate burden this patient population places on mental health services.
